# Concordance of three alternative gestational age assessments for pregnant women from four African countries: A secondary analysis of the MIPPAD trial

**DOI:** 10.1371/journal.pone.0199243

**Published:** 2018-08-06

**Authors:** Samantha Rada, Jutta Gamper, Raquel González, Ghyslain Mombo-Ngoma, Smaïla Ouédraogo, Mwaka A. Kakolwa, Rella Zoleko-Manego, Esperança Sevene, Abdunoor M. Kabanywanyi, Manfred Accrombessi, Valérie Briand, Michel Cot, Anifa Vala, Peter G. Kremsner, Salim Abdulla, Achille Massougbodgi, Arsénio Nhacolo, John J. Aponte, Eusébio Macete, Clara Menéndez, Michael Ramharter

**Affiliations:** 1 Center for Public Health, Department of Epidemiology, Medical University of Vienna, Vienna, Austria; 2 Institut für Medizinische Informationsverarbeitung Biometrie und Epidemiologie, Ludwig-Maximilians University, Munich, Germany; 3 Department of Medicine I, Division of Infectious Diseases and Tropical Medicine, Medical University of Vienna, Vienna, Austria; 4 Center for Medical Statistics, Informatics and Intelligent Systems, Medical University of Vienna, Vienna, Austria; 5 Barcelona Centre for International Health Research, (CRESIB, Hospital Clínic-Universitat de Barcelona), ISGlobal, Barcelona Institute for Global Health, Barcelona, Spain; 6 Manhiça Health Research Center (CISM), Manhiça, Mozambique; 7 Centre de Recherches Médicales de Lambaréné (CERMEL), Albert Schweitzer Hospital, Lambaréné, Gabon; 8 Institute of Tropical Medicine, University of Tübingen, Tübingen, Germany; 9 German Center for Infectious Diseases (DZIF), Tübingen, Germany; 10 Department of Parasitology, Université des Sciences de la Santé (USS), Libreville, Gabon; 11 Faculté des Sciences de la Santé (FSS), Université d’Aboméy Calavi, Cotonou, Benin; 12 Institut de Recherche pour le Développement (IRD), Paris, France; 13 Ministère de la Santé, Burkina Faso; 14 Ifakara Health Institute (IHI), Dodoma, Tanzania; 15 Ngounie Medical Research Centre, Fougamou, Gabon; 16 Université René Descartes, Paris, France; 17 Department of Tropical Medicine, Bernhard Nocht Institute for Tropical Medicine and University Medical Center Hamburg-Eppendorf, Hamburg, Germany; Univesity of Iowa, UNITED STATES

## Abstract

**Background:**

At times, ultrasound is not readily available in low resource countries in Africa for accurate determination of gestational age, so using alternative methods is pivotal during pregnancy. These assessments are used to aid the risk analysis for an infant and management strategies for premature delivery, if necessary. Currently, date of last menstrual period, fundal height measurements, and the New Ballard Score are commonly used in resource-limited settings. However, concordance of these measures is unknown for sub-Saharan Africa. We obtained data from an open-label randomized controlled trial, to assess the concordance of these alternative assessment methods. The purpose of our study was to determine the agreement between these alternative methods when used in sub-Saharan African populations.

**Methods:**

A total of 4,390 pregnant women from Benin, Gabon, Mozambique and Tanzania were included in our analysis. The assessment methods compared were: 1) reported last menstrual period, 2) symphysis-fundal height measurement, and 3) the New Ballard Score. The Bland-Altman method and intraclass correlation coefficient (ICC) were used to test the degree of agreement. Survival range gestational age, used as an inclusion criterion for further analysis, was from 22 to 44 weeks.

**Findings:**

Plots showed a lack of agreement between methods and the 95% limits of agreement too wide to be clinically useful. ICC = 0.25 indicated poor agreement. A post-hoc analysis, restricted from 32 to 42 weeks, was done to check for better agreement in this near-term population. The plots and ICC = 0.16 still confirmed poor agreement.

**Conclusion:**

The alternative assessments do not result in comparable outcomes and discrepancies are far beyond the clinically acceptable range. Last menstrual period should not be used as the only estimator of gestational age. In the absence of reliable early ultrasound, symphysis-fundal height measurements may be most useful during pregnancy for fetal risk assessment and the New Ballard Score after delivery as a confirmation of these estimations and for further neonatal management. However, promotion of portable ultrasound devices is required for accurate assessment of gestational age in sub-Sahara Africa.

## Introduction

Gestational age, the duration of pregnancy that begins with conception, is a necessity to measure in the pregnancy and delivery process for the establishment of optimal antenatal and postnatal management and care plans for the mother and infant. Precise estimates of gestational age are used to identify risks the fetus or neonate can succumb to such as pre-term birth (< 37 weeks) and fetal growth restriction which both contribute to the high numbers of low birth weight, especially in low-resource settings [1–3]. In developed countries early abdominal ultrasound examination, generally performed in the first trimester, is used as the gold standard for the determination of gestational age, however in low-resource settings, where this technology is inaccessible or unavailable, healthcare workers must rely on other methods to determine gestational age [4].

Alternative methods for assessing gestational age are reported last menstrual period (LMP), symphysis-fundal height measurement (SFH) and the New Ballard Score [4, 5]. The preferred method of assessment varies during pregnancy with LMP being most frequently used early in pregnancy, SFH around 20 weeks of gestation and the New Ballard Score used after delivery. Last menstrual period gestational age, taken early on in pregnancy, is an interval originating from the first date of the last normal menstrual cycle relying upon the recall of the woman [6]. Symphysis-Fundal height measurement, by bimanual palpation, is the distance from the top of the symphysis pubis (pubic bone) to the top of the uterine fundus and is most accurately measured around 20 weeks of gestation when the fundus is above the symphysis [5]. The New Ballard Score, a set of procedures [S1 File] requiring 12 inputs assessing physical and neuromuscular maturity of the neonate to determine its gestational age, is only performed postnatally up to 96 hours after birth [7]. Since it is taken postnatally, this estimation of gestational age cannot be used for risk assessment before birth, but rather for neonatal healthcare. It has been shown that these three methods constitute reasonable measurements for gestational age when compared to ultrasound and that they are also acceptable methods when assessing gestational age in low-resource settings [4,5,7,8,9]. The purpose of this study was to determine if the three alternative methods for estimating gestational age were in agreement with one another for pregnant women from four African countries participating in a randomized controlled trial for malaria prevention.

## Methods

### Study area and population

Gestational age data was taken from an open-label, randomized, three-arm trial that took place from 2009 to 2013 that compared intermittent preventive treatments against malaria in pregnancy (IPTp) [10,11] in the sub-Saharan countries of Benin, Gabon, Mozambique, and Tanzania [12,13]. The primary study was approved by the Ethics Committees from the Hospital Clínic of Barcelona (Spain), the Comité Consultatif de Déontologie et d'Éthique (CCDE) from the Institut de Recherche pour le Développement (IRD, in France). All local regulatory authorities and the following Ethics Review Committees from each malaria endemic country, also approved the study: Comité d’ Ethique de l’Université Abomey Calavi (Benin), Comité d’Ethique Régional Indépendant de Lambaréne (Gabon), Comité Nacional de Bioética para a Saúde (Mozambique), Departamento Farmacéutico (Mozambique), Institutional Review Board (Tanzania), National Institute for Medical Research Review Board(Tanzania), Tanzania Food and Drug Association (Tanzania). The primary trial was conducted under the provisions of the Declaration of Helsinki and in accordance with Good Clinical Practices guidelines set up by the WHO and by the International Conference on Harmonization.

### Enrollment and assessments

Women that were enrolled in the study were included after signing written informed consent and with the following inclusion criteria: permanent residence in the study area, negative HIV-testing at recruitment, absence of history of allergy to sulfa drugs or mefloquine, absence of history of renal, hepatic, psychiatric or neurological diseases and gestational age ≤ 28 weeks. The women selected after the screening process constituted the cohort for this analysis on the concordance of the three gestational age assessments: last menstrual period, symphysis-pubis fundal height and the New Ballard Score. Estimates of gestational age in weeks that were used for the analysis were assessed at the mother’s first antenatal care visit (LMP and SFH) and at birth (New Ballard Score).

Mother IDs and infant IDs containing these data were matched accordingly considering that not every study participant gave birth. A stillbirth was defined as the death of the baby ≥ 28 weeks after the mother becomes pregnant and spontaneous abortion (or miscarriage) as the unexpected natural death of the fetus or embryo before 28 weeks gestation. Ectopic gestation is defined as a complication in pregnancy when a fertilized egg is implanted outside of the uterus potentially leading to life-threatening complications in early pregnancy. While stillbirths were included in our analysis, spontaneous abortions and ectopic pregnancies were not. Twins were also considered in our dataset because of the impossibility to know in advance whether a pregnancy will be a twin pregnancy during gestation if ultrasound is not used. Since the New Ballard Score would be different for each twin, mothers’ gestational age measurements were observed for each of the infants that were born and the LMP and SFH values from the first twin were also used for the second twin. Study participants that had only one gestational age recorded throughout pregnancy and delivery were excluded from this comparative analysis. The live birth of an infant is possible between 22 to 44 weeks gestational age [14, 15] and this survival range was therefore used as inclusion criterion for this analysis.

### Data management and statistical analysis

Agreement of the three methods was assessed by the Bland-Altman method and by the intraclass correlation coefficient (ICC). For the Bland-Altman analysis, pairwise comparisons were created as follows: LMP versus SFH, LMP versus the New Ballard score and SFH versus the New Ballard score. Subjects were included in each pairwise group if they had at least two gestational ages recorded and at least one out of those two gestational ages were within the survival range (22 to 44 weeks). In these pairwise groups, means and differences between each method were calculated for every study participant and plotted against each other with the means between methods on the horizontal axis and the differences between methods on the vertical axis. The mean differences as well as the 95% limits of agreement, calculated by the mean of the differences (*đ)* ± 1.96 × standard deviation, were also added to the Bland-Altman plots.

For the intraclass correlation coefficient analysis, subjects were included if at least one out of the three methods for each individual subject contained a gestational age within the survival range. We used a linear mixed model to estimate the variance of the random subject effect and residual variance. The ICC was calculated by the given subject variance divided by the total variance. Interpretation of ICC agreement is as follows: poor agreement ≤ 0.40, fair agreement 0.40–0.59, good agreement 0.60–0.74 and excellent agreement ≥ 0.75. Data analysis was performed using SPSS statistical software (IBM, 22.0.0.0), R (3.3.1) and Microsoft Excel (14.0.0).

## Results

**Fig 1:**
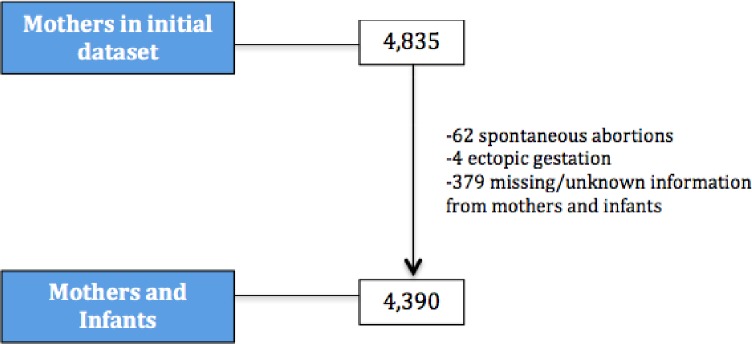
Flowchart of mothers and infants used in study.

Overall, 4,390 pregnant women with a mean age of 25 ± 6 (range, 13–49) years were analyzed. Baseline characteristics of the mothers and infants are shown in Table 1. Out of 4,835, the total number of study participants from the primary study, 62 women were excluded from our analysis because of spontaneous abortion, four were excluded for ectopic gestation and 379 women were excluded due to missing or unknown information (Fig 1).

**Table 1:** Baseline characteristics of mothers and infants.

**Study Participants (Mothers)**	**N = 4,390**
**Age (years)**^**a**^	25 ± 6
**Weight (kg)**^**a**^	59.7 ± 11.1
**Height (cm)**^**a**^	157.9 ± 7.5
**MUAC (mm)**^**a**^	26.5 ± 3.6
**Country**^**b**^	
** Benin**	1,130 (25.7)
** Gabon**	1,029 (23.4)
** Mozambique**	1,142 (26.1)
** Tanzania**	1,089 (24.8)
**Can read**^**a**^	
** Yes**	2,993 (68.2)
** No**	1,397 (31.8)
**Can write**^**b**^	
** Yes**	3,017 (68.7)
** No**	1,373 (31.3)
**Infants**	N = 4,390
**Birth Weight (kg)**^**a**^	2.9 ± 0.5
**Length (cm)**^**a**^	48.3 ± 3.8
**Head Circumference (cm)**^**a**^	33.7 ±2.3

^a^ Arithmetic mean ± standard deviation

^b^ *n* (percentage)

MUAC; middle upper arm circumference

Gestational ages that were estimated with the New Ballard Score in Tanzania were excluded from all parts of the analysis because estimations were not assessed correctly according to protocol (n = 1,062). LMP gestational ages (n = 1,300) were missing because women were not able to recall their date of last menstruation. Descriptive statistics for each of the three methods are shown in Table 2**.** LMP shows the widest range of estimated gestational age followed by the New Ballard Score and SFH showing a similar range.

**Table 2:** Descriptive statistics of each method.

	N	Missing	Min. (weeks)	Max. (weeks)	Mean (weeks)	Median (weeks)
**Last Menstrual Period**	3,090	1,300	9	66	38.6	39
**Symphysis- Fundal Height**	4,269	121	21	48	39.3	39
**New Ballard Score**	2,928	1,441	13	50	38.8	38

The number of subjects in each pairwise comparison group that were used for each Bland-Altman plot, after excluding those with only one gestational age recorded was 3,038 (LMP vs. SFH), 1,795 (LMP vs. New Ballard Score), and 2,870 (SFH vs. New Ballard Score), respectively. Those with two gestational ages recorded were excluded only if both estimations were outside of the survival range of 22 to 44 weeks. Each study participant had at least one gestational age within the survival range, from the two estimations recorded.

**Fig 2:**
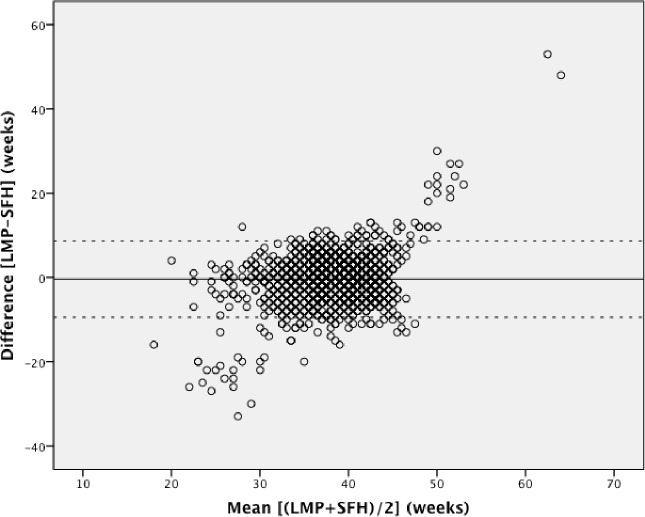
Bland-Altman plot: Last menstrual period versus symphysis fundal height. Mean difference (solid line) and 95% limits of agreement (dotted line).

**Fig 3:**
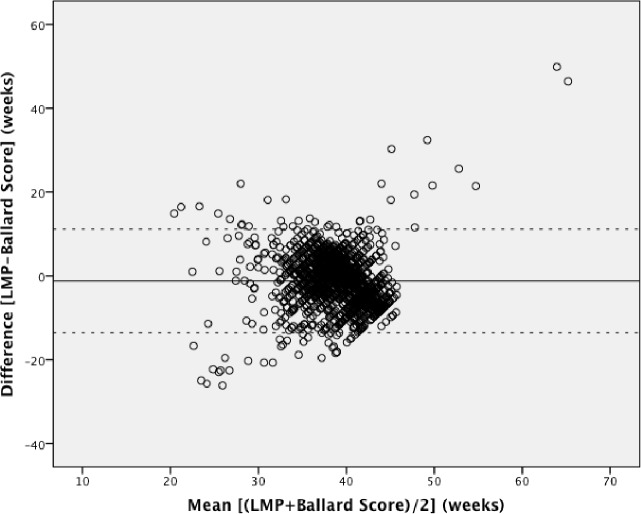
Bland-Altman plot: Last menstrual period versus the New Ballard Score. Mean difference (solid line) and 95% limits of agreement (dotted line).

**Fig 4:**
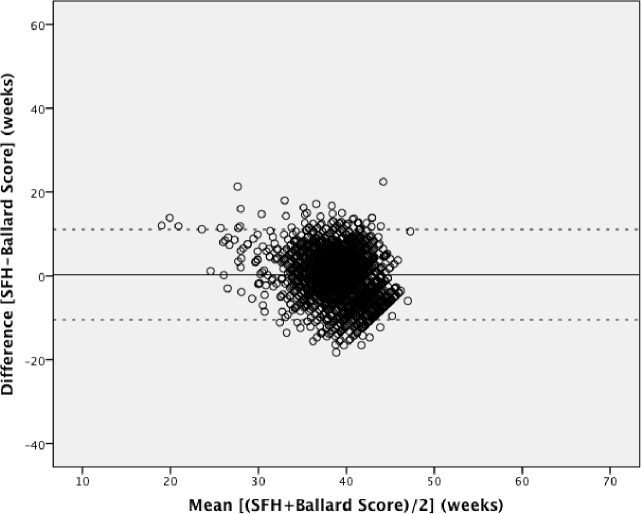
Bland-Altman plot: Symphysis fundal height versus the New Ballard Score. Mean difference (solid line) and 95% limits of agreement (dotted line).

Pairwise Bland-Altman plots with the 95% limits of agreement show the means between methods plotted on the horizontal axis and differences between methods plotted on the vertical axis (Figs 2–4). The numerical results associated with these plots are shown in Table 3. Examining the plots visually, between 32 to 42 weeks gestational age, most estimates lie within the 95% limits of agreement. However, outside of this 32–42 week range, the data show a wide variation throughout the mean gestational age. When mean gestational age is less than 32 or greater than 42 weeks, the difference increases. Table 3 shows that the 95% limits of agreement result in a particularly large range of differences and the 95% CIs around the mean differences for all methods do not contain the line of equality (y = 0; no difference between methods). LMP shows under- and over-estimation of gestational age. LMP gestational age estimations had the greatest range of implausible values with 9 weeks being the minimum estimation and 66 weeks being the highest. Although each Bland-Altman plot shows lack of agreement, the highest consistency is observed in Fig 4.

**Table 3:** Numerical results within Bland-Altman plots (weeks).

Pairwise Comparison	Mean Difference (solid line)	Lower 95% CI of Mean Difference	Upper 95% CI of Mean Difference	Standard Deviation	Lower LOA (dotted line)	Upper LOA (dotted line)
LMP vs. SFH	-0.40	-0.56	-0.23	4.62	-9.46	8.66
LMP vs. New Ballard	-1.24	-1.53	-0.95	6.31	-13.61	11.13
SFH vs. New Ballard	0.31	0.11	0.51	5.49	-10.45	11.07

LOA; level of agreement; Mean difference ± 1.96 * standard deviation.

All 4,390 subjects were used for the ICC analysis since each subject had one gestational age within the survival range. In the linear mixed model the ICC, which is the subject variance divided by the total variance, was 0.25. Thus, due to this low ICC value, the agreement between the methods is categorized as “poor” confirming the lack of agreement between methods.

### Post-hoc analysis

**Fig 5:**
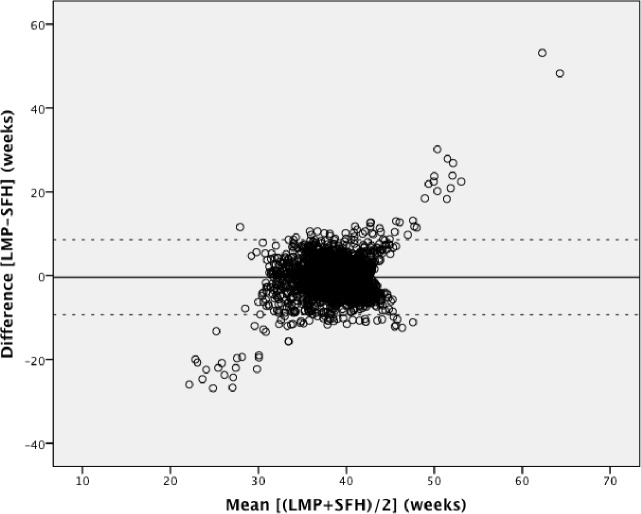
Post-hoc Bland-Altman plot: Last menstrual period versus symphysis fundal height. Mean difference (solid line) and 95% limits of agreement (dotted line).

**Fig 6:**
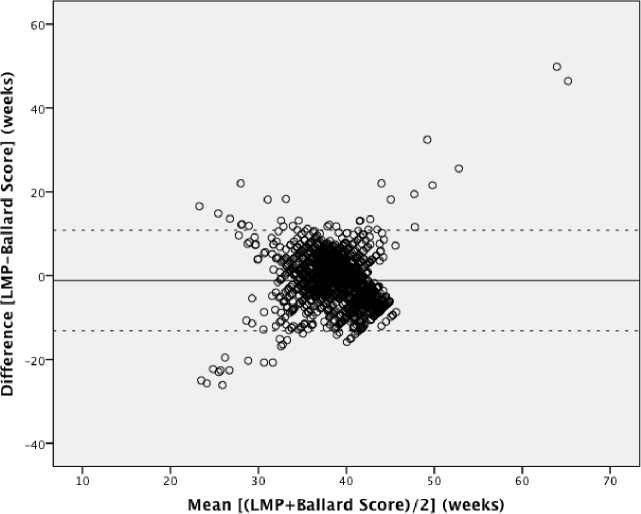
Post-hoc Bland-Altman plot: Last menstrual period versus the New Ballard Score. Mean difference (solid line) and 95% limits of agreement (dotted line).

**Fig 7:**
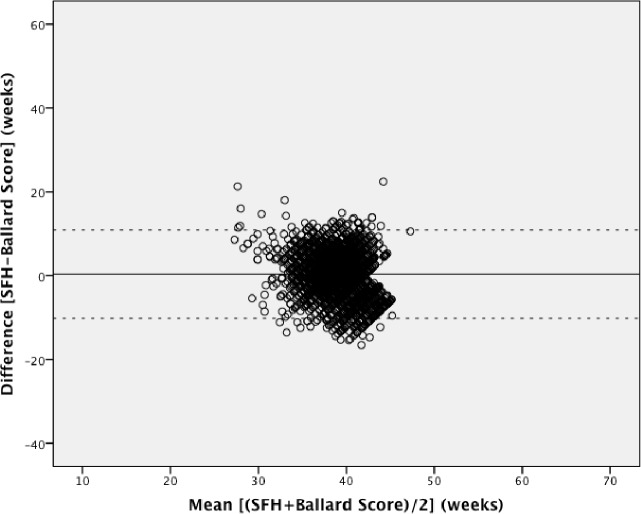
Post-hoc Bland-Altman plot: Last menstrual period versus the New Ballard Score. Mean difference (solid line) and 95% limits of agreement (dotted line).

The three methods showed no agreement in the planned comparative analysis; therefore a post-hoc analysis restricted to 32–42 weeks gestational age was performed to explore if there was a better agreement in this near term population. This was to see if gestational ages below 32 weeks or above 42 weeks influenced disproportionally the concordance of these methods. After restriction, 162 pairs were excluded from LMP vs. SFH (n = 2876), 70 pairs from LMP vs. New Ballard Score (n = 1725) and 109 pairs from SFH vs. New Ballard Score (n = 2761). Bland-Altman and ICC analyses were repeated for the sub-analysis. Subjects were included if they had at least one gestational age within the 32–42 week range. Figs 5–7 show the Bland Altman plots with the 95% limits of agreement and Table 4 contains the numerical results associated with these plots. The analysis shows that there is still poor concordance between methods. This lack of agreement is again confirmed with the ICC = 0.16.

**Table 4:** Numerical results within post-hoc Bland-Altman plots (weeks).

Pairwise Comparison	Mean Difference (solid line)	Lower 95% CI of Mean Difference	Upper 95% CI of Mean Difference	Standard Deviation	Lower LOA (dotted line)	Upper LOA (dotted line)
LMP vs. SFH	-0.38	-0.55	-0.21	4.55	-9.30	8.54
LMP vs. New Ballard	-1.15	-1.44	-0.86	6.11	-13.13	10.83
SFH vs. New Ballard	0.33	0.13	0.54	5.38	-10.21	10.87

LOA; level of agreement; Mean difference ± 1.96 * standard deviation.

## Discussion

In this study the relationship between three alternative gestational age assessment methods was assessed to determine their degree of concordance. An accurate measurement of gestational age is required for adequate care of the mother and infant during pregnancy and after birth to estimate and prevent risks. Although the three methods measured the same estimated outcome, there were visible differences between them.

We used the specified survival range because evidence shows that a live birth can still occur as early as 22 weeks and as late as 44 weeks [14, 15]. By keeping those subjects with at least one gestational age within this range, we risked including subjects with other implausible estimations, but this allowed us to test true differences between methods in this clinically relevant population. Due to the nature of the assessments by physical examination and history of last menstrual period, there is bound to be errors within these assessments. Our findings showed noticeable differences with a considerable proportion of the estimations far from being clinically useful, which resulted in the variation of differences and means in our Bland-Altman plots. The Bland-Altman plots assume that the mean difference is constant across values, but this may not be the case in our situation. Outputs of t-test on the differences gave us statistically significant p-values (p <0.001), indicating that there were significant differences between each method. As also seen and described in Giavarina et al., the line of equality (y = 0) did not fall within the 95% confidence interval of the mean difference in any of our plots, which indicates a significant systematic difference [16]. Similarly, the ICC from the initial analysis and post-hoc analysis (0.25 and 0.16, respectively) indicates that the reliability of the gestational age estimations is quite low when comparing between methods for each individual study participant. From the post-hoc analysis it was therefore confirmed that the estimations between 22 to 32 weeks and 42 to 44 weeks were not the underlying reason for this poor concordance.

The 95% limits of agreement in both the planned and post-hoc analyses were far too large to be clinically useful. At an individual level, a discrepancy of ± one week in respective measurements would arguably be within the limit of tolerance for not being harmful to the mother or infant, but an estimation beyond that could potentially undermine its usefulness for a proper risk assessment or management. Symphysis-fundal height versus the New Ballard Score Bland-Altman plot showed the smallest range of means within the limits of agreements, but still a large range of differences. The observed discrepancies in each plot were far beyond the clinically acceptable range. When examining the Bland-Altman plots, the measurements are more dispersed when comparing LMP against any of the other two methods (Figs 2 and 3), than what is seen in Fig 4. This over dispersion is most likely related to the inclusion of LMP values that are clearly not realistic with survival and are due to misreporting by the pregnant women. However, by keeping these unrealistic values, the clinical usefulness of LMP reporting could be assessed in a real world setting. It was therefore concluded that LMP cannot be used as the only assessment for assessing gestational age in these populations. This recall of information for the LMP method is prone to error, particularly in situations of high illiteracy or of late enrollment for antenatal care [5, 17]. This may not play an important role in our study, considering more than half of the study participants could read and write (68.2% and 68.7%, respectively), but inaccuracy can be seen by the improbable values seen in the dataset. LMP may also be difficult to assess due to irregular menstrual cycles varying in duration, lactational amenorrhea, bleeding early in pregnancy, or hormonal contraceptive use preceding conception [9]. As shown in our study, LMP had a tendency to overestimate gestational age if used alone, but in previous studies it was considered to be clinically useful if confirmed with ultrasound [4, 6, 9, 18, 19]. The same can be said about SFH, although in a previous study, SFH has shown more agreement with ultrasound assessment than LMP [9]. In a study by Jehan et al., it was suggested that symphysis-fundal height may be favored over last menstrual period particularly in low-resource settings where ultrasound is unavailable [9]. Based on previous work, the New Ballard Score seems to be the most accurate and reliable method when determining gestational age when compared to other methods [4, 5, 17].

Due to the absence of a gold standard test, it is unknown which method is objectively better in terms of getting a precise estimation of gestational age. Although comparing to a gold standard was not the aim of the study, it became a significant limitation for the comparison of the alternative methods, deeming it difficult to interpret our findings when creating the Bland-Altman plots. Although we did not have a gold standard to compare, future studies should evaluate the alternative assessments as done in our study, and categorize comparisons for pre-term, term and post-term pregnancies. Although the large sample size is a strength in our study, data for each method was not collected for each subject which led to a considerable rate of exclusion and missing data. Despite the fact that 4,390 women were included in the analysis, only 3,038 women, 2,870 women and 1,795 women were used in the pairwise comparisons LMP vs. SFH, SFH vs. New Ballard, LMP vs. New Ballard, respectively. This rate of exclusion can be seen as a limitation in this study.

Despite the absence of concordance between the three gestational age assessments, the use of alternative gestational age assessment is useful to guide clinical management. Based on the particularly poor correlation of LMP with the other two gestational age assessments, it may be useful to recommend the use of the SFH method over the LMP method taken throughout different intervals of gestation and the New Ballard Score after delivery.

Ultimately, these data show that no reliable method for gestational age assessment exists in this setting and that early ultrasound is required for accurate determination of gestational age. Accurate ultrasound assessments become increasingly available with the use of affordable, portable devices when operated by well-trained technicians. In addition of gestational age assessment these devices are important tools for the diagnosis of obstetric complications in resource-limited regions [20]. Based on the presented data it is therefore recommended that affordable ultrasound devices be introduced to developing countries, to be used as a reference standard in conjunction with available alternative methods for the proper evaluation of gestational age and the clinical management of pregnancy.

## Supporting information

S1 File The New Ballard Score sheet. (PDF)

S1 Table Descriptive statistics of each method from GABON. (PDF)

S2 Table Descriptive statistics of each method from MOZAMBIQUE. (PDF)

S3 Table Descriptive statistics of each method from BENIN. (PDF)

S4 Table Descriptive statistics of each method from TANZANIA. (PDF)

S1 Fig Bland-Altman plot without Tanzania: Last menstrual period versus symphysis fundal height. Mean difference (solid line) and 95% LOA (dotted line).
(PDF)

S2 Fig Bland-Altman plot without Tanzania: Last menstrual period versus New Ballard Score. Mean difference (solid line) and 95% LOA (dotted line).
(PDF)

S3 Fig Bland-Altman plot without Tanzania: Symphysis fundal height versus New Ballard Score. Mean difference (solid line) and 95% LOA (dotted line).
(PDF)

S4 Fig Bland-Altman plot GABON: Last menstrual period versus symphysis fundal height. Mean difference (solid line) and 95% LOA (dotted line).
(PDF)

S5 Fig Bland-Altman plot GABON: Last menstrual period versus New Ballard Score. Mean difference (solid line) and 95% LOA (dotted line).
(PDF)

S6 Fig Bland-Altman plot GABON: Symphysis fundal height versus New Ballard Score. Mean difference (solid line) and 95% LOA (dotted line).
(PDF)

S7 Fig Bland-Altman plot MOZAMBIQUE: Last menstrual period versus symphysis fundal height. Mean difference (solid line) and 95% LOA (dotted line).
(PDF)

S8 Fig Bland-Altman plot MOZAMBIQUE: Last menstrual period versus New Ballard Score. Mean difference (solid line) and 95% LOA (dotted line).
(PDF)

S9 Fig Bland-Altman plot MOZAMBIQUE: Symphysis fundal height versus New Ballard Score. Mean difference (solid line) and 95% LOA (dotted line).
(PDF)

S10 Fig Bland-Altman plot BENIN: Last menstrual period versus symphysis fundal height. Mean difference (solid line) and 95% LOA (dotted line).
(PDF)

S11 Fig Bland-Altman plot BENIN: Last menstrual period versus New Ballard Score. Mean difference (solid line) and 95% LOA (dotted line).
(PDF)

S12 Fig Bland-Altman plot BENIN: Symphysis fundal height versus New Ballard Score. Mean difference (solid line) and 95% LOA (dotted line).
(PDF)
