# Supplementary material for: Concordance of three alternative gestational age assessments for pregnant women from four African countries: A secondary analysis of the MIPPAD trial
Source: PLoS One. 2018 Aug 6;13(8):e0199243. doi: 10.1371/journal.pone.0199243 (PMC6078285; doi:10.1371/journal.pone.0199243)
Supplement: S1 File — (PDF) [file pone.0199243.s001.pdf]

S1 File. The New Ballard Score sheet

The New Ballard Score

www.ballardscore.com

NEUROMUSCULAR MATURITY

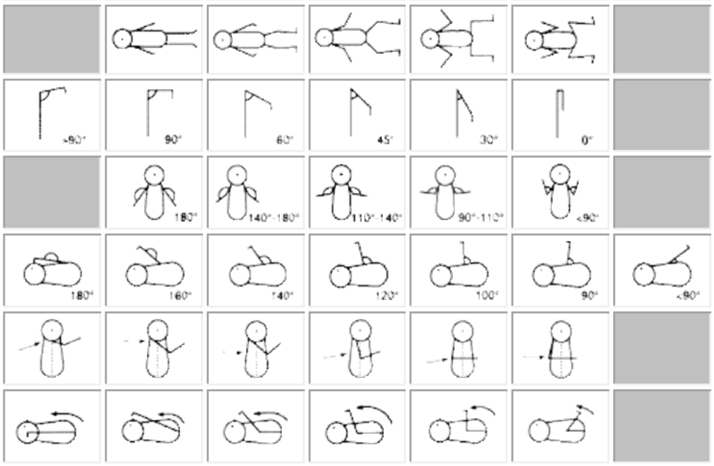

SIGN	SCORE -1	0	1	2	3	4	5	SIGN SCORE
Posture								
Square Window	>90°	90°	60°	45°	30°	0°		
Arm Recoil		180°	140°-180°	110°-140°	90°-110°	<90°		
Popliteal Angle	180°	160°	140°	120°	100°	90°	<90°	
Scarf Sign								
Heel To Ear								
							TOTAL NEUROMUSCULAR SCORE	

MATURITY RATING

TOTAL SCORE	WEEKS
-10	20
-5	22
0	24
5	26
10	28
15	30
20	32
25	34
30	36
35	38
40	40
45	42
50	44

SIGN	SCORE -1	0	1	2	3	4	5	SIGN SCORE
Skin	Sticky, friable, transparent	gelatinous, red, translucent	smooth pink, visible veins	superficial peeling &/or rash, few veins	cracking, pale areas, rare veins	parchment, deep cracking, no vessels	leathery, cracked, wrinkled	
Lanugo	none	sparse	abundant	thinning	bald areas	mostly bald		
Plantar Surface	heel-toe 40-50mm: -1 <40mm: -2	>50 mm no crease	faint red marks	anterior transverse crease only	creases ant. 2/3	creases over entire sole		
Breast	imperceptable	barely perceptable	flat areola no bud	stippled areola 1-2 mm bud	raised areola 3-4 mm bud	full areola 5-10 mm bud		
Eye / Ear	lids fused loosely: -1 tightly: -2	lids open pinna flat stays folded	sl. curved pinna; soft; slow recoil	well-curved pinna; soft but ready recoil	formed & firm instant recoil	thick cartilage ear stiff		
Genitals (Male)	scrotum flat, smooth	scrotum empty, faint rugae	testes in upper canal, rare rugae	testes descending, few rugae	testes down, good rugae	testes pendulous, deep rugae		
Genitals (Female)	clitoris prominent & labia flat	prominent clitoris & small labia minora	prominent clitoris & enlarging minora	majora & minora equally prominent	majora large, minora small	majora cover clitoris & minora		
							TOTAL PHYSICAL MATURITY SCORE	

Gestation by Dates

	weeks

Birth date	Hour	
		am<br>pm

APGAR	1 min	5min

Scoring

Gest. Age by Maturity Rating	________weeks
Time of Exam	Date__________<br>am<br>Hour______ pm
Age at Exam	_________hours

Signature of Examiner

____

____

M.D. / R.N.

References :

Ballard JL, Khoury JC, Wedig K, *et al*: New Ballard Score, expanded to include extremely premature infants. *J Pediatrics* 1991; 119:417-423.

http://ballardscore.com/Pages/ScoreSheet.aspx
