# Supplementary material for: Concordance of three alternative gestational age assessments for pregnant women from four African countries: A secondary analysis of the MIPPAD trial
Source: PLoS One. 2018 Aug 6;13(8):e0199243. doi: 10.1371/journal.pone.0199243 (PMC6078285; doi:10.1371/journal.pone.0199243)
Supplement: S2 Table — (PDF) [file pone.0199243.s003.pdf]

S2 Table. Descriptive statistics of each method from MOZAMBIQUE

	N	Missing	Min. (weeks)	Max. (weeks)	Mean (weeks)	Median (weeks)
**Last Menstrual Period**	913	228	11	65	38.4	39.1
**Symphysis-fundal Height**	1115	26	22	44	38.1	38.0
**New Ballard Score**	994	149	13	45	36.9	37.0
