# Supplementary material for: Concordance of three alternative gestational age assessments for pregnant women from four African countries: A secondary analysis of the MIPPAD trial
Source: PLoS One. 2018 Aug 6;13(8):e0199243. doi: 10.1371/journal.pone.0199243 (PMC6078285; doi:10.1371/journal.pone.0199243)
Supplement: S3 Table — (PDF) [file pone.0199243.s004.pdf]

S3 Table. Descriptive statistics of each method from BENIN

	N	Missing	Min. (weeks)	Max. (weeks)	Mean (weeks)	Median (weeks)
**Last Menstrual Period**	163	970	19	49	38.0	38.9
**Symphysis-fundal Height**	1102	32	25	47	39.4	40.0
**New Ballard Score**	977	158	23	49	37.7	38.0
