# Supplementary material for: Concordance of three alternative gestational age assessments for pregnant women from four African countries: A secondary analysis of the MIPPAD trial
Source: PLoS One. 2018 Aug 6;13(8):e0199243. doi: 10.1371/journal.pone.0199243 (PMC6078285; doi:10.1371/journal.pone.0199243)
Supplement: S4 Table — (PDF) [file pone.0199243.s005.pdf]

S4 Table. Descriptive statistics of each method from TANZANIA

	N	Missing	Min. (weeks)	Max. (weeks)	Mean (weeks)	Median (weeks)
**Last Menstrual Period**	1039	50	9	64	39.4	39.9
**Symphysis-fundal Height**	1066	21	22	40	37.1	37.0
**New Ballard Score**	21	1041	-	-	-	-

- New Ballard Score values excluded from analysis.
