# Supplementary material for: Concordance of three alternative gestational age assessments for pregnant women from four African countries: A secondary analysis of the MIPPAD trial
Source: PLoS One. 2018 Aug 6;13(8):e0199243. doi: 10.1371/journal.pone.0199243 (PMC6078285; doi:10.1371/journal.pone.0199243)
Supplement: S11 Fig — Mean difference (solid line) and 95% LOA (dotted line). (PDF) [file pone.0199243.s016.pdf]

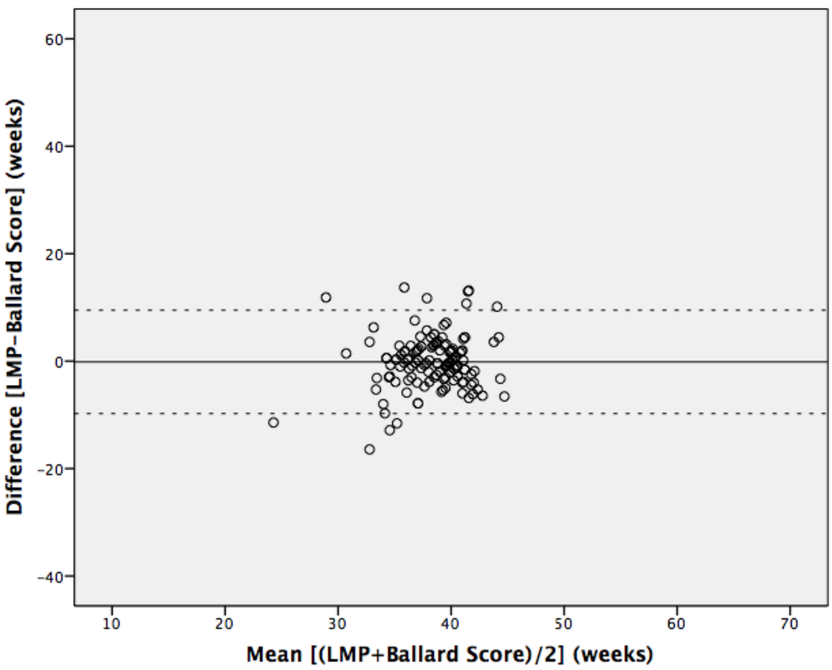

S11 Fig. Bland-Altman plot BENIN: Last menstrual period versus New Ballard Score.
Mean difference (solid line) and 95% LOA (dotted line).
